# Evaluation of tri‐ponderal mass index in predicting metabolic associated fatty liver disease in obese children

**DOI:** 10.1111/ped.70205

**Published:** 2025-09-17

**Authors:** Yavuz Özer, Ersin Ulu, Emine Yurdakul Ertürk

**Affiliations:** ^1^ Department of Pediatric Endocrinology Zeynep Kamil Women and Children's Diseases Training and Research Hospital Istanbul Turkey; ^2^ Cerrahpasa Medical Faculty, Department of Neonatology Istanbul University‐Cerrahpasa Istanbul Turkey; ^3^ Faculty of Medicine, Department of Pediatrics Ordu University Ordu Turkey

**Keywords:** MAFLD, obesity, pediatric metabolic risk, tri‐Ponderal mass index

## Abstract

**Background:**

This study aimed to evaluate the effectiveness of the tri‐ponderal mass index (TMI) in identifying metabolic dysfunction‐associated fatty liver disease (MAFLD) in obese children and adolescents.

**Methods:**

A total of 238 obese children and adolescents were included in the study. Evaluations included anthropometric measurements, liver function tests, lipid parameters, fasting glucose, insulin, and HOMA‐IR. Logistic regression analysis was performed to determine the predictive value of TMI for MAFLD.

**Results:**

MAFLD was diagnosed in 55.5% of participants. TMI values were significantly higher in the MAFLD group compared to those without MAFLD (mean TMI: 21.01 ± 3.05 vs. 20.14 ± 2.90, *p* = 0.026). In logistic regression analyses, male sex was an independent predictor in both models (OR = 3.10, *p* = 0.003 in the BMI‐SDS model; OR = 2.63, *p* = 0.008 in the TMI model). HOMA‐IR (OR = 1.10, *p* = 0.042; OR = 1.11, *p* = 0.029) and ALT (OR = 1.04, *p* = 0.002; OR = 1.05, *p* = 0.002) were also significant in both models. TMI was not independently associated with MAFLD (*p* = 0.249). TMI showed positive correlations with age, weight‐SDS, BMI‐SDS, fasting insulin, HbA1c, HOMA‐IR, and the grade of hepatosteatosis.

**Conclusion:**

While TMI values were higher in patients with MAFLD, TMI was not an independent predictor of MAFLD in obese children. Male sex, HOMA‐IR, and ALT were consistently associated with MAFLD, suggesting greater predictive value. These findings suggest that TMI may have limited utility as a screening tool for MAFLD in pediatric clinical practice.

## INTRODUCTION

Obesity has emerged as one of the most significant public health challenges of the 21st century and is recognized as a non‐communicable chronic disease. The rising prevalence of obesity among children and adolescents is a growing global concern.^1^ As a major independent risk factor, obesity contributes to the development of numerous chronic conditions, including cardiovascular diseases, type 2 diabetes, hypertension, non‐alcoholic fatty liver disease (NAFLD), and certain cancers.^2^

Given the increasing burden of pediatric obesity, accurately assessing body composition is critical for identifying individuals at risk of metabolic complications. Various methods, ranging from basic anthropometric measurements to advanced imaging techniques, are utilized for this purpose. Among them, body mass index (BMI) remains the most widely used due to its simplicity and practicality. However, BMI has notable limitations: it does not differentiate between fat mass and lean mass, nor does it account for fat distribution. Additionally, in children and adolescents, BMI is influenced by age, sex, and pubertal development, which reduces its reliability in assessing adiposity and metabolic risk.^3^, ^4^ To overcome these limitations, alternative anthropometric indices that more accurately reflect body fat composition have been proposed. In recent years, the Tri‐Ponderal Mass Index (TMI) has gained attention as a potentially superior tool for evaluating body composition in pediatric populations.^5^, ^6^, ^7^ Furthermore, TMI has demonstrated utility in screening for insulin resistance^8^ and hepatosteatosis^9^ in children and adolescents.

A growing body of evidence underscores the association between obesity and metabolic complications, particularly hepatic steatosis.^10^, ^11^ A meta‐analysis reported a NAFLD prevalence of 34.2% among obese children and adolescents attending obesity clinics.^12^ Recently, the term NAFLD has been revised to metabolic dysfunction‐associated fatty liver disease (MAFLD) to better reflect its metabolic underpinnings. MAFLD is characterized by excessive hepatic fat accumulation in individuals with metabolic and cardiovascular risk factors, independent of other liver diseases.^13^ In children, hepatic steatosis is often asymptomatic, leading to delayed diagnosis and an increased risk of progressive liver disease in adulthood. Therefore, early detection and prevention of hepatosteatosis should be integral components of pediatric obesity management.^10^

Given the increasing prevalence of both obesity and MAFLD in the pediatric population, there is a critical need for reliable, practical, and cost‐effective methods to assess body composition and predict metabolic risk. This study aims to investigate the relationship between TMI and MAFLD in obese children and adolescents, evaluating the potential of TMI as a practical tool for identifying those at risk for metabolic complications.

## MATERIALS AND METHODS

This retrospective study included children and adolescents aged 6 to 18 years who were diagnosed with obesity at the pediatric endocrinology outpatient clinic between September 1, 2022, and November 30, 2024. Demographic, anthropometric, laboratory, and ultrasonographic data were extracted from medical records. Simple obesity was defined as a body mass index (BMI) above the 95th percentile for age, in the absence of hepatosteatosis on ultrasonography and with normal liver function tests. Pediatric MAFLD was diagnosed based on the presence of hepatosteatosis confirmed by imaging, accompanied by at least one cardiometabolic risk factor (CMRF). Participants with congenital anomalies, syndromic obesity, endocrine disorders, psychiatric disorders, or any known cause of hepatic steatosis (e.g., viral hepatitis, prior parenteral nutrition, specific medications, autoimmune liver disease, or metabolic liver disease) were excluded.

Physical examination and pubertal staging were performed by the same pediatric endocrinologist to ensure consistency. Weight was measured using a calibrated digital scale with 100 g sensitivity, and height was measured with a stadiometer (SECA, model 220, Hamburg, Germany). Obesity was classified according to BMI values exceeding the 95th percentile based on national reference data for Turkish children and adolescents.^14^ BMI was calculated as weight (kg) divided by height squared (m^2^), and the TMI was calculated as weight (kg) divided by height cubed (m^3^).

The study protocol was approved by the Ethics Committee of Zeynep Kamil Women and Children's Diseases Training and Research Hospital (Approval No: 42, dated August 7, 2024). The study adhered to the principles outlined in the Declaration of Helsinki and Good Clinical Practice guidelines. Due to its retrospective design, the requirement for informed consent was waived.

Pediatric MAFLD was defined as intrahepatic fat accumulation with at least one of the following: overweight or obesity, prediabetes or type 2 diabetes, or evidence of metabolic dysregulation — defined by the presence of at least two age‐ and sex‐specific metabolic risk factors, including elevated waist circumference, hypertension, hypertriglyceridemia, low high‐density lipoprotein cholesterol (HDL‐C) levels, or impaired fasting glucose.^13^

Laboratory analyses, including serum glucose, alanine aminotransferase (ALT), aspartate aminotransferase (AST), and lipid profiles, were performed using standard enzymatic methods with a Roche Cobas 8000 c 702 analyzer. Cutoff values for laboratory parameters were defined based on pediatric clinical guidelines and national reference data. ALT >40 IU/L and AST >34 IU/L were considered elevated. Fasting blood glucose (FBG) ≥100 mg/dL was defined as impaired. HbA1c ≥5.7% was used as a threshold for elevated glycemia. Low‐density lipoprotein‐cholesterol (LDL‐C) >130 mg/dL, HDL‐C < 40 mg/dL, and total cholesterol >200 mg/dL were considered abnormal. For triglycerides, triglyceride >100 mg/dL was used for children aged 6–9 years and triglyceride >130 mg/dL for those aged 10–18 years. Insulin resistance was assessed using the Homeostasis Model Assessment for Insulin Resistance (HOMA‐IR), calculated as:

$$\text{HOMA}-\mathrm{IR}=\left[\text{fasting insulin}\;\left(\mathrm{\mu U}/\mathrm{mL}\right)\times \text{fasting glucose}\;\left(\mathrm{mg}/\mathrm{dL}\right)\right]/405$$

Insulin resistance was defined based on HOMA‐IR thresholds specific to Turkish children, with cutoff values of >2.22 for prepubertal girls, >2.67 for prepubertal boys, >3.82 for pubertal girls, and >5.22 for pubertal boys.^15^

Liver ultrasonography was performed by the same radiologist using a Toshiba Alpio 500 device. Hepatic steatosis was graded based on liver echogenicity as follows: grade 1 (mild), grade 2 (moderate), and grade 3 (severe).^16^

### Statistical analysis

Statistical analyses were conducted using Jamovi software (version 2.3.21). The Kolmogorov–Smirnov test was applied to assess the normality of data distribution. Categorical variables were expressed as frequencies and percentages, while continuous variables were presented as either mean ± standard deviation (for normally distributed data) or median with interquartile range [IQR] (for non‐normally distributed data). Comparisons between two groups were made using the Student's *t*‐test for normally distributed continuous variables and the Mann–Whitney U test for non‐normally distributed variables. Chi‐square tests were applied for categorical data comparisons. Spearman's rank correlation analysis was performed to investigate the relationships between TMI and demographic, anthropometric, and biochemical parameters. To explore the predictive value of BMI‐SDS and TMI for MAFLD, we constructed two separate binomial logistic regression models, each including one of the indices along with other covariates. The results were reported as odds ratios (ORs) with 95% confidence intervals (CIs). Additionally, receiver operating characteristic (ROC) curve analysis was employed to determine the area under the curve (AUC), cutoff values, sensitivity, and specificity of selected markers for predicting MAFLD. A *p*‐value <0.05 was considered statistically significant.

## RESULTS

A total of 238 obese pediatric and adolescent patients were included in the study, with 132 (55.5%) classified in the MAFLD group and 106 (44.5%) in the non‐MAFLD group. Among those with MAFLD, hepatic steatosis was classified by ultrasonography as grade 1 (mild) in 84 participants (63.7%), grade 2 (moderate) in 37 participants (28.0%), and grade 3 (severe) in 11 participants (8.3%). The median age of participants was 13.9 years (IQR: 11.1–15.6), with no significant difference between groups (*p* = 0.162).

The proportion of males was significantly higher in the MAFLD group than in the non‐MAFLD group (43.9% vs. 19.8%, *p* < 0.001), with male sex associated with a 3.17‐fold increased risk of MAFLD (OR: 3.17, 95% CI: 1.76–5.71). No significant difference was observed in pubertal status between the two groups (*p* = 0.594).

Anthropometric assessments revealed that weight SDS, BMI‐SDS, and TMI were significantly higher in the MAFLD group (*p* = 0.003, *p* = 0.021, and *p* = 0.026, respectively), while height SDS did not differ significantly (*p* = 0.113). However, when analyzed by sex, mean TMI was 21.00 ± 3.12 in males and 20.40 ± 2.88 in females, with no statistically significant difference observed between the groups (*p* = 0.299).

Regarding laboratory findings, ALT and AST levels were significantly elevated in patients with MAFLD (both *p* < 0.001). Fasting insulin, HOMA‐IR, and HbA1c levels were also significantly higher in the MAFLD group (*p* = 0.001, *p* < 0.001, and *p* = 0.001, respectively). No significant difference was observed in fasting blood glucose levels (*p* = 0.276). In lipid profile analysis, HDL‐C levels were significantly lower (*p* < 0.001) and triglyceride levels were significantly higher (*p* = 0.008) among MAFLD patients, while LDL‐C and total cholesterol levels did not differ significantly between groups (*p* = 0.510 and *p* = 0.684, respectively).

Demographic, clinical, and laboratory characteristics of the study population are presented in Table 1.

**TABLE 1:** Demographic, anthropometric, and laboratory characteristics of patients with and without MAFLD.

		Total (*n* = 238)	Non‐MAFLD (*n* = 106)	MAFLD (*n* = 132)	*p*‐value
Age (years)^a^		13.9 (11.1–15.6)	13.3 (10.0–15.4)	13.9 (11.6–15.7)	0.162*
Gender *n*, (%)	Male	79 (33.2)	21 (19.8)	58 (43.9)	<0.001**
	Female	159 (66.8)	85 (80.2)	74 (56.1)	
Pubertal Status *n*, (%)	Prepubertal	37 (15.5)	15 (14.2)	22 (16.7)	0.594**
	Pubertal	201 (84.5)	91 (85.8)	110 (83.3)	
Weight SDS^b^		3.18 ± 1.09	2.94 ± 0.94	3.36 ± 1.16	0.003***
Height SDS^b^		0.35 ± 1.18	0.21 ± 1.19	0.46 ± 1.15	0.113***
BMI‐SDS^b^		2.89 ± 0.66	2.78 ± 0.61	2.98 ± 0.68	0.021***
TMI^b^		20.62 ± 3.01	20.14 ± 2.90	21.01 ± 3.05	0.026***
ALT (IU/L)^a^		21.0 (15.0–35.0)	17.0 (13.0–25.0)	27.5 (18.0–40.8)	<0.001*
AST (IU/L)^a^		21.0 (21.0–28.5)	19.0 (17.0–24.0)	25.0 (18.0–32.0)	<0.001*
FBG (mg/dL)^a^		92 (87–99)	92 (87–98)	93 (88–100)	0.276*
Fasting insulin (IU/mL)^a^		26.2 (16.2–37.0)	23.0 (14.9–31.5)	31.2 (19.6–41.0)	0.001*
HbA1c (%)^a^		5.5 (5.3–5.6)	5.4 (5.2–5.6)	5.5 (5.3–5.7)	0.001*
HOMA‐IR^a^		6.07 (3.48–8.38)	4.94 (3.20–7.31)	6.76 (4.46–9.52)	<0.001*
LDL‐C (mg/dL)^b^		92.1 ± 27.0	90.8 ± 28.0	93.2 ± 26.3	0.510***
HDL‐C (mg/dL)^b^		46.2 ± 9.8	48.8 ± 9.9	44.2 ± 9.3	<0.001***
Total cholesterol (mg/dL)^b^		162.2 ± 31.1	161.3 ± 29.1	163.0 ± 32.7	0.684***
Triglyceride (mg/dL)^b^		127.0 ± 54.7	116.4 ± 42.6	135.6 ± 61.7	0.008***

*Note*: In the MAFLD group, weight SDS, BMI‐SDS, TMI, serum ALT, AST, fasting insulin, HbA1c, HOMA‐IR, and triglyceride levels were higher, while serum HDL‐C levels were lower compared to the non‐MAFLD group.

Abbreviations: ALT, alanine aminotransferase; AST, aspartate aminotransferase; BMI, body mass index; FBG, fasting blood glucose; HbA1c, hemoglobin A1c; HDL‐C, high‐density lipoprotein‐cholesterol; HOMA‐IR, homeostatic model assessment of insulin resistance; LDL‐C, low‐density lipoprotein‐cholesterol; MAFLD, metabolic associated fatty liver disease; SDS, standard deviation score; TG, triglyceride; TMI, tri‐ponderal mass index.

* Mann–Whitney U test.

** Chi‐square test.

*** Student's *t*‐test.

^a^ Values represent median (25th‐75th percentile).

^b^ Values represent mean ± standard deviation.

Binomial logistic regression analyses were performed using two separate models to evaluate the independent associations of BMI‐SDS and TMI with MAFLD. In Model 1, including BMI‐SDS, male sex (OR = 3.10, *p* = 0.003), HOMA‐IR (OR = 1.10, *p* = 0.042), and ALT (OR = 1.04, *p* = 0.002) were identified as significant predictors. BMI‐SDS showed a borderline association (*p* = 0.052), and HDL‐C approached significance (*p* = 0.062) (Table 2). In Model 2, which included TMI, male sex (OR = 2.63, *p* = 0.008), HOMA‐IR (OR = 1.11, *p* = 0.029), and ALT (OR = 1.05, *p* = 0.002) remained significant, whereas TMI itself was not (*p* = 0.249). HDL‐C again showed a borderline *p*‐value (0.070) (Table 3). Model performance was similar across models (Pseudo R^2^ = 0.21 and 0.20, respectively; *p* < 0.001 for both).

**TABLE 2:** Binomial logistic regression model 1 including BMI‐SDS as a predictor of MAFLD in obese children.

	β	Standard error	Odds ratio	*p*‐value	95% confidence interval	
					Lower	Upper
Age	0.05972	0.07712	1.0615	0.439	0.912	1.230
Gender (male)	1.13062	0.37977	3.0976	0.003	1.472	6.520
Pubertal status (pubertal)	−0.53570	0.58514	0.5853	0.360	0.186	1.840
BMI‐SDS	0.55384	0.28510	1.7399	0.052	0.995	3.040
FBG	0.00468	0.01012	1.0047	0.644	0.985	1.020
HOMA‐IR	0.09326	0.04581	1.0977	0.042	1.003	1.200
ALT	0.04372	0.01420	1.0447	0.002	1.016	1.070
AST	−0.00557	0.01228	0.9944	0.650	0.971	1.020
HDL‐C	−0.03690	0.01980	0.9638	0.062	0.927	1.000
Total Cholesterol	0.00221	0.00648	1.0022	0.733	0.989	1.020
Triglyceride	0.00114	0.00411	1.0011	0.781	0.993	1.010

*Note*: Model 1 performance: Pseudo *R* ^2^ = 0.21, *p* < 0.001. In the binomial logistic regression model 1 including BMI‐SDS, male gender, elevated HOMA‐IR, and increased ALT levels were identified as independent predictors of MAFLD.

Abbreviations: ALT, alanine aminotransferase; AST, aspartate aminotransferase; BMI, body mass index; FBG, fasting blood glucose; HDL‐C, high‐density lipoprotein–cholesterol; HOMA‐IR, homeostatic model assessment of insulin resistance; SDS, standard deviation score.

**TABLE 3:** Binomial logistic regression model 2 including TMI as a predictor of MAFLD in obese children.

	β	Standard error	Odds ratio	*p*‐value	95% confidence interval	
					Lower	Upper
Age	0.09549	0.07312	1.1002	0.192	0.902	1.226
Gender (male)	0.96731	0.36366	2.6308	0.008	1.290	5.370
Pubertal status (pubertal)	−0.62105	0.58152	0.5374	0.286	0.172	1.680
TMI	0.06571	0.05697	1.0679	0.249	0.955	1.190
FBG	0.00558	0.00989	1.0056	0.572	0.986	1.030
HOMA‐IR	0.10021	0.04594	1.1054	0.029	1.010	1.210
ALT	0.04461	0.01414	1.0456	0.002	1.017	1.080
AST	−0.00673	0.01229	0.9933	0.584	0.970	1.020
HDL‐C	−0.03616	0.01993	0.9645	0.070	0.928	1.000
Total cholesterol	0.00269	0.00644	1.0027	0.676	0.990	1.020
Triglyceride	0.00100	0.00412	1.0010	0.808	0.993	1.010

*Note*: Model 2 performance: Pseudo *R* ^2^ = 0.20, *p* < 0.001. In the binomial logistic regression model 2 including BMI‐SDS, male gender, elevated HOMA‐IR, and increased ALT levels were identified as independent predictors of MAFLD.

Abbreviations: ALT, alanine aminotransferase; AST, aspartate aminotransferase; HDL‐C, high‐density lipoprotein cholesterol; FBG, fasting blood glucose; HOMA‐IR, homeostatic model assessment of insulin resistance; TMI, tri‐ponderal mass index.

A correlation analysis was performed to evaluate the relationship between TMI and various demographic, anthropometric, and biochemical parameters (Table 4). TMI was positively correlated with age (ρ = 0.239, *p* < 0.001), weight SDS (ρ = 0.578, *p* < 0.001), BMI‐SDS (ρ = 0.817, *p* < 0.001), fasting insulin (ρ = 0.183, *p* = 0.005), HbA1c (ρ = 0.141, *p* = 0.040), HOMA‐IR (ρ = 0.156, *p* = 0.016), and the ultrasound‐based grade of hepatic steatosis (ρ = 0.229, *p* < 0.001). No significant correlation was observed between TMI and ALT, AST, fasting blood glucose, LDL‐C, total cholesterol, or triglyceride levels (*p* > 0.05). Additionally, a weak negative correlation was found between TMI and HDL‐C, but it was not statistically significant.

**TABLE 4:** Correlation between TMI and clinical parameters.

	Rho	*p*‐value
Age	0.239	<0.001
Weight SDS	0.578	<0.001
BMI‐SDS	0.817	<0.001
ALT	0.097	0.138
AST	0.049	0.455
FBG	0.097	0.141
Fasting insulin	0.183	0.005
HbA1c	0.141	0.040
HOMA‐IR	0.156	0.016
LDL‐C	0.003	0.961
HDL‐C	−0.098	0.136
Total Cholesterol	−0.038	0.561
Triglyceride	−0.021	0.746
Grade of hepatosteatosis	0.229	<0.001

*Note*: TMI was positively correlated with age, weight SDS, BMI‐SDS, fasting insulin, HbA1c, HOMA‐IR, and the grade of hepatosteatosis.

Abbreviations: ALT, alanine aminotransferase; AST, aspartate aminotransferase; BMI, body mass index; FBG, fasting blood glucose; HbA1c, hemoglobin A1c; HDL‐C, high‐density lipoprotein cholesterol; HOMA‐IR, homeostatic model assessment of insulin resistance; LDL‐C, low‐density lipoprotein cholesterol; SDS, standard deviation score; TG, triglyceride; TMI, tri‐ponderal mass index.

**FIGURE 1:**
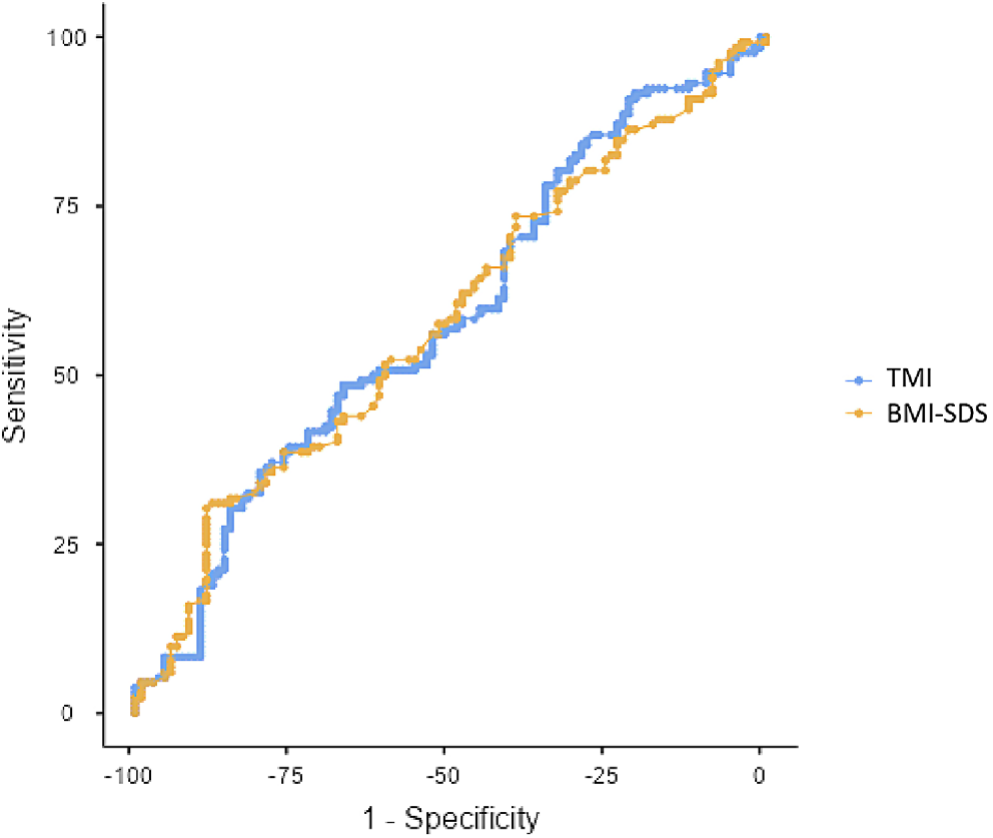
ROC curve for TMI and BMI‐SDS to predict MAFLD.

The diagnostic performance of TMI and BMI‐SDS for identifying MAFLD was assessed through ROC curve analysis (Table 5 and Figure 1). Both indices demonstrated poor discriminatory ability. For TMI, the optimal cutoff value was 22.16, with an AUC of 0.584, sensitivity of 35.61%, and specificity of 80.19%. For BMI‐SDS, the cutoff value was 3.42, with an AUC of 0.581, sensitivity of 30.3%, and specificity of 88.68%. There was no significant difference between the AUC values of TMI and BMI‐SDS (*p* = 0.918).

**TABLE 5:** ROC curve analysis for TMI and BMI‐SDS in predicting MAFLD.

Index	AUC	SE	95% CI (AUC)	Cut‐off	Sensitivity	Specificity	PPV	NPV	*p*‐value
TMI	0.584	0.022	0.541–0.627	22.16	35.61%	80.19%	69.12%	50%	0.918*
BMI‐SDS	0.581	0.021	0.539–0.623	3.42	30.3%	88.68%	76.92%	50.54%	

*Note*: Both indices demonstrated poor discriminative ability, with AUC values around 0.58. The DeLong test revealed no statistically significant difference between the AUCs.

Abbreviations: PPV, positive predictive value; NPV, negative predictive value; AUC, area under the curve; BMI‐SDS, Body Mass Index‐standard deviation score; TMI, Tri‐Ponderal Mass Index.

* Difference between the AUCs, using the DeLong test.

## DISCUSSION

In this study, we investigated the relationship between the TMI and MAFLD in obese children and adolescents. In our cohort, the prevalence of MAFLD was 55.5%, whereas a previous study by Trochimczyk et al.^17^ reported a higher prevalence of 70% among children with obesity. Our results demonstrated that children with MAFLD exhibited significantly higher adiposity indices, greater insulin resistance, and a more unfavorable lipid profile compared to their non‐MAFLD counterparts. Furthermore, logistic regression analysis identified male sex, elevated HOMA‐IR, and higher ALT levels as independent predictors of MAFLD. Although BMI‐SDS did not reach statistical significance, its near‐significant association suggests that it may achieve significance in studies with larger sample sizes. Importantly, TMI was not found to be an independent predictor of MAFLD.

The relationship between adiposity indices and MAFLD is well‐documented, with BMI and weight‐based measures frequently used in clinical practice. However, recent studies have suggested that TMI may be a more stable and age‐independent marker for assessing body fat percentage in pediatric populations.^6^, ^18^ Prior research has proposed TMI as a potential predictor of MAFLD.^9^, ^19^ Consistent with these findings, we observed significant correlations between TMI and several metabolic parameters, including insulin resistance markers and the grade of hepatosteatosis. However, despite these associations, ROC curve analysis demonstrated limited discriminatory power for both TMI and BMI‐SDS (AUC ≈ 0.58), suggesting that while they reflect adiposity, they may not be sufficiently robust as standalone screening tools for clinical decision‐making. Similarly, Umaro et al. reported that the waist‐to‐height ratio was a superior predictor of NAFLD compared to BMI‐SDS and TMI. Moreover, Yetim et al.^20^ found no significant difference in TMI values between children with and without NAFLD when assessed by magnetic resonance imaging (MRI). These findings suggest that although TMI alone may not be sufficient for MAFLD screening, its combination with metabolic indicators such as ALT, HOMA‐IR, and HDL‐C could increase its clinical relevance, particularly within a multi‐marker predictive model for hepatic steatosis in pediatric obesity.

Our findings also reinforce previous reports indicating a higher prevalence of NAFLD among male children.^9^, ^12^, ^20^, ^21^, ^22^ This sex‐based difference may stem from variations in body fat distribution, hormonal influences, and metabolic regulation. The 3.17‐fold increased risk of MAFLD observed in male patients underscores the importance of gender‐specific risk assessment and intervention strategies. Furthermore, insulin resistance appears to play a central role in the pathogenesis of MAFLD, as evidenced by the strong association between HOMA‐IR and MAFLD risk in our study. Similarly, Yetim et al.^20^ also identified elevated HOMA‐IR as an independent predictor of NAFLD. Additionally, Trochimczyk et al.^17^ demonstrated that novel insulin resistance indices, such as the triglyceride‐glucose index (TyG) and the triglyceride‐to‐HDL‐C ratio (TG/HDL‐C), outperformed HOMA‐IR in predicting MAFLD in the pediatric population. Elevated ALT levels further reflect underlying hepatic injury and metabolic dysfunction, correlating with hepatic steatosis and inflammation.^10^ Lipid profile alterations, particularly lower HDL‐C levels, were another key finding in our study. Dyslipidemia is a hallmark of metabolic syndrome and has been implicated in MAFLD progression. Moreover, the observed lower HDL‐C levels among MAFLD patients align with existing evidence suggesting that HDL‐C may exert protective effects against hepatic steatosis.^9^, ^10^ These metabolic abnormalities highlight the need for early identification and targeted lifestyle interventions focusing on weight reduction, improved insulin sensitivity, and lipid profile optimization to prevent and manage MAFLD in children.

In this study, hepatic steatosis was evaluated by conventional ultrasonography. Although US is a widely used, accessible, and noninvasive imaging modality, it has inherent limitations, including operator dependency and an inability to differentiate between simple steatosis, steatohepatitis, and fibrosis. Moreover, compared to advanced imaging methods such as MRI or elastography, US offers limited sensitivity and lacks quantitative assessment capabilities. In particular, it may underestimate mild hepatic steatosis when compared to these more advanced modalities. Nevertheless, due to its low cost, accessibility, and practicality, US remains the preferred screening tool, particularly in pediatric clinical settings.^23^

Recent international consensus has proposed the use of the terms MASLD (Metabolic dysfunction‐associated steatotic liver disease) and MASH (Metabolic dysfunction‐associated steatohepatitis) to better reflect the underlying metabolic dysfunction and reduce disease‐related stigma.^24^ Although our study utilized the MAFLD definition consistent with the prevailing terminology at the time of data collection, we acknowledge this nomenclature update and encourage future research to adopt the current standards.

Despite providing valuable insights, this study has several limitations. First, the cross‐sectional design precludes the establishment of causal relationships between TMI and MAFLD. Second, the diagnosis of MAFLD relied on imaging findings rather than histological confirmation, which remains the gold standard for diagnosis. Finally, the sample size, although reasonable, may not have been large enough to detect certain subtle associations. Future longitudinal studies with larger cohorts and biopsy‐based diagnoses are needed to better define the predictive value of TMI and its integration with other metabolic markers.

In conclusion, our findings suggest that TMI is correlated with metabolic risk factors in obese children and adolescents but has limited standalone predictive value for MAFLD. Male sex, insulin resistance, and elevated ALT levels emerged as key determinants of MAFLD, emphasizing the need for comprehensive metabolic evaluation in at‐risk pediatric populations. Future research should explore whether integrating TMI with additional metabolic or inflammatory biomarkers could improve the identification of children at risk for MAFLD.

## AUTHOR CONTRIBUTIONS

Y.Ö., E.U., and E.Y.E. contributed to the conception and design of the study. Y.Ö. and E.Y.E. were responsible for participant enrollment, data management, and study monitoring. Statistical analysis was performed by Y.Ö. and E.U. Y.Ö. drafted the manuscript. All authors critically reviewed the manuscript and approved the final version for submission.

## FUNDING INFORMATION

This research received no specific grant from any funding agency in the public, commercial, or not‐for‐profit sectors.

## CONFLICT OF INTEREST STATEMENT

The authors declare no conflicts of interest.

## ETHICS STATEMENT

The study was conducted in accordance with the principles of the Declaration of Helsinki. Ethical approval was obtained from the Ethics Committee of Zeynep Kamil Women and Children's Diseases Training and Research Hospital (Approval No: 42, dated August 7, 2024). Due to its retrospective design, the requirement for informed consent was waived.

## Data Availability

The data that support the findings of this study are available from the corresponding author upon reasonable request.
